# Orthopaedic diagnostic pitfalls in fibroblast growth factor 23-mediated hypophosphatemic rickets/osteomalacia in fibrous dysplasia/McCune-Albright syndrome: Two burosumab-treated cases

**DOI:** 10.1016/j.bonr.2026.101945

**Published:** 2026-08-31

**Authors:** Tomoo Nakagawa, Jungo Imanishi, Ayako Koda, Taisuke Matsuyama, Kenji Sato, Asako Yamamoto, Kentaro Matsui, Yoshinobu Watanabe, Hirotaka Kawano

**Affiliations:** a Department of Orthopaedic Surgery, Teikyo University School of Medicine, 2-11-1 Kaga, Itabashi-ku, Tokyo, 173-8605, Japan; b Trauma and Reconstruction Center, Teikyo University Hospital, 2-11-1 Kaga, Itabashi-ku, Tokyo, 173-8605, Japan; c Department of Orthopaedic Surgery, Higashi Kawaguchi Hospital, 2-10-8 Higashi Kawaguchi, Kawaguchi-shi, Saitama, 333-0801, Japan; d Trauma Center, Toranomon Hospital, 2-2-2 Toranomon, Minato-ku, Tokyo 105-8470, Japan.; e Department of Radiology, Teikyo University School of Medicine, 2-11-1 Kaga, Itabashi-ku, Tokyo, 173-8605, Japan

**Keywords:** Fibrous dysplasia, McCune-Albright syndrome, Fibroblast growth factor 23, Hypophosphatemia, Osteomalacia, Rickets, Burosumab

## Abstract

Fibrous dysplasia/McCune-Albright syndrome (FD/MAS) can obscure fibroblast growth factor 23 (FGF23)-mediated hypophosphatemic rickets/osteomalacia. We report two FD/MAS patients with recurrent fractures, hypophosphatemia, elevated FGF23, and serial imaging showing overlooked rachitic changes years before diagnosis. Burosumab improved serum phosphate levels after dose adjustment and was associated with mobility gains in Case 1 and increased growth velocity with physeal normalization in Case 2. Age-appropriate phosphate assessment and FGF23 testing are warranted when orthopaedic findings suggest impaired mineralization.

## Highlights

• FD/MAS can obscure FGF23-mediated rickets/osteomalacia.
• Serial imaging can reveal overlooked rachitic changes.
• Recurrent low-energy fractures should prompt phosphate testing.
• Age-specific phosphate interpretation is critical in children.
• Burosumab improved phosphate status and physeal morphology.

## 1 Introduction

Fibrous dysplasia/McCune-Albright syndrome (FD/MAS) is a rare mosaic disorder caused by postzygotic activating *GNAS* variants (Spencer et al., 2019). Recent nationwide registry data estimate the prevalence of FD/MAS to be approximately 61 per 1,000,000 persons (Meier et al., 2024). In bone, constitutive Gsα activation disrupts osteogenic differentiation and replaces normal bone and marrow with expansile fibro-osseous tissue, leading to pain, deformity, pathological fracture, and functional impairment (Javaid et al., 2019; Boyce and Collins, 2020). Orthopaedic management often focuses on fracture stabilization, deformity, and rehabilitation, but FD/MAS may also have a metabolic component that aggravates skeletal fragility.

Fibrous dysplasia lesions can produce excess fibroblast growth factor 23 (FGF23), causing renal phosphate wasting, reduced 1,25-dihydroxyvitamin D production, and impaired mineralization (Riminucci et al., 2003; Bhattacharyya et al., 2012). In orthopaedic practice, the key diagnostic step is to recognize when recurrent fractures or rickets/osteomalacia-like imaging findings should be followed by biochemical evaluation. The 2015 Japanese diagnostic criteria and flowchart for rickets and osteomalacia start from clinical or radiographic suspicion and use serum phosphate as an early branch point (Fukumoto et al., 2015). In hypophosphatemic patients, the flowchart then recommends FGF23 testing to identify FGF23-related hypophosphatemic rickets/osteomalacia. This framework is especially relevant in FD/MAS, where dysplastic bone lesions can obscure persistent hypophosphatemia.

FGF23-mediated hypophosphatemic rickets/osteomalacia is a potentially treatable condition, but it may be overlooked in FD/MAS when fractures, deformity, or functional decline are attributed to dysplastic bone alone. We report two patients with FD/MAS-associated FGF23-mediated hypophosphatemic rickets/osteomalacia diagnosed during orthopaedic care. This report focuses on the orthopaedic clues that prompted biochemical evaluation and led to recognition of a coexisting, modifiable phosphate and mineralization disorder.

## 2 Cases

In both cases, serum intact FGF23 concentrations were measured by chemiluminescent enzyme immunoassay (CLEIA) at SRL, Inc. (Tokyo, Japan).

### 2.1 Case 1: Transition from pediatric to adult care in a 22-year-old man with FD/MAS

**Fig. 1:**
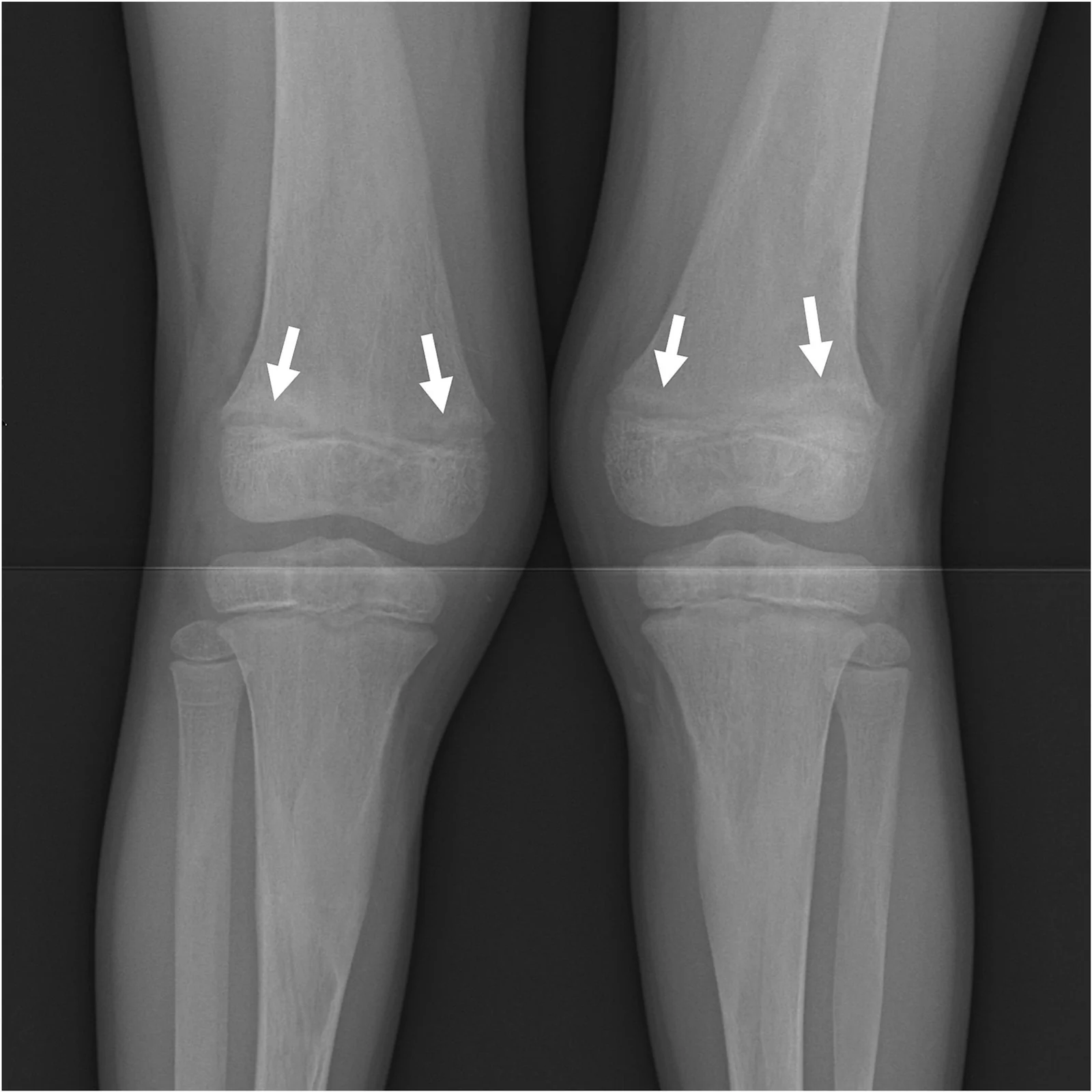
Previously unrecognized rachitic changes in Case 1 at age 8. Anteroposterior radiograph of bilateral knees showing distal femoral metaphyseal widening, flaring, and fraying with physeal irregularity. Arrows indicate metaphyseal irregularity adjacent to the distal femoral physes. These findings were retrospectively consistent with rachitic changes.

A 22-year-old man with FD/MAS was referred during transition from pediatric to adult orthopaedic care. He had been diagnosed with McCune-Albright syndrome at age 5 after right hip pain led to the recognition of polyostotic fibrous dysplasia. The diagnosis was supported by café-au-lait macules, gonadotropin-independent precocious puberty, and detection of an activating *GNAS* p.Arg201Cys (R201C; CGT>TGT) variant in DNA extracted from peripheral blood. The analysis was performed by PCR followed by restriction enzyme digestion and Sanger sequencing; the variant allele fraction was not quantified. During reassessment at age 22, radiographs obtained at age 8 were retrospectively reviewed and showed bilateral distal femoral metaphyseal widening, flaring, and fraying with physeal irregularity, consistent with previously unrecognized rachitic changes (Fig. 1).

**Fig. 2:**
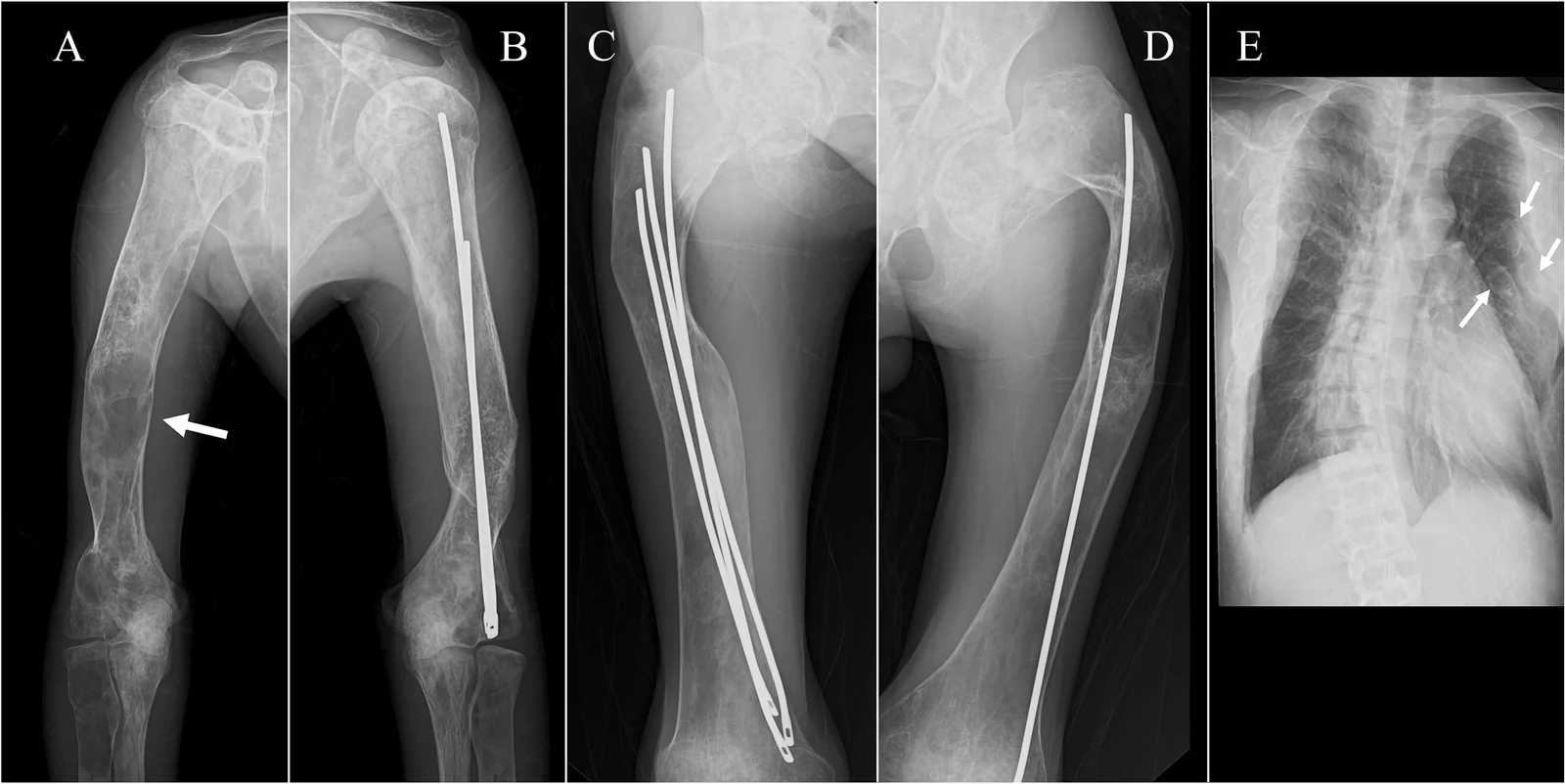
Polyostotic fibrous dysplasia and previous fracture treatment sites in Case 1 at transition to adult care. Representative radiographs obtained at age 22 show extensive polyostotic fibrous dysplasia involving the bilateral humeri (A, B), bilateral femora (C, D), and ribs/thoracic skeleton (E). Previous Ender nail fixation is visible in the left humerus and both femora, and severe proximal femoral varus deformity with a shepherd's crook configuration is present. The arrow in A indicates a recent fracture site with associated cystic change; arrows in E indicate multiple old rib fracture sites.

**Fig. 3:**
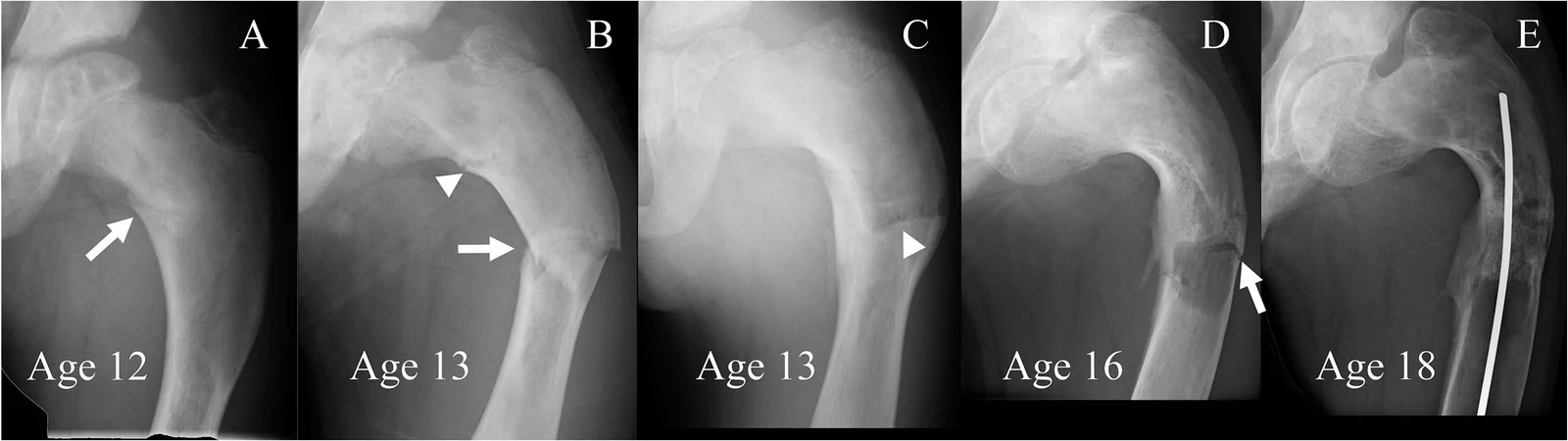
**Long-term orthopaedic course of the left femur in Case 1.** Serial radiographs show recurrent left femoral pathological fractures, a cortical radiolucency (Looser zone), progressive deformity, and subsequent internal fixation during long-term follow-up. (A) First pathological fracture at age 12. (B) Second pathological fracture at age 13, with a Looser zone in the proximal femur. (C) Persistent Looser zone at age 13 after the second fracture. (D) Third pathological fracture at age 16. (E) Radiograph at age 18 showing the status after repeated pathological fractures, progressive deformity, and internal fixation. Arrows indicate the pathological fracture sites in A, B, and D; arrowheads indicate the Looser zones in B and C.

At age 12, he sustained a left subtrochanteric femoral pathological fracture. Thereafter, he experienced repeated fractures involving both proximal femora and both proximal humeri, treated with casting or Ender nail fixation. He remained ambulatory until approximately age 15, but subsequently became wheelchair-dependent because of recurrent fractures, deformity, and pain. At age 22, he sustained a right distal humeral fracture during low-energy activity, prompting referral to the adult orthopaedic service. Radiographs showed extensive polyostotic FD involving the bilateral humeri, femora, and thoracic skeleton, multiple previous fracture treatment sites, and severe proximal femoral varus deformity with a shepherd's crook configuration (Fig. 2). Serial radiographs demonstrated recurrent femoral pathological fractures, progressive deformity, and repeated fixation over long-term orthopaedic follow-up (Fig. 3).

Because of recurrent low-energy fractures, FD/MAS, and functional decline, blood testing was performed and showed serum phosphate 2.4 mg/dL (adult male laboratory reference interval, 2.7–4.6 mg/dL), alkaline phosphatase 1533 U/L (38–113 U/L; IFCC method), bone-specific alkaline phosphatase 472 μg/L (3.7–20.9 μg/L), calcium 9.0 mg/dL (8.8–10.1 mg/dL), intact PTH 85 pg/mL (10–65 pg/mL), and 25-hydroxyvitamin D 5.5 ng/mL (vitamin D deficiency, defined as <20 ng/mL; Okazaki et al., 2017). Because hypophosphatemia was present, FGF23 was measured in accordance with the diagnostic pathway for rickets/osteomalacia and was 144 pg/mL (laboratory reference interval, 19.9–52.9 pg/mL). The inappropriately elevated FGF23 level in the setting of hypophosphatemia was consistent with the diagnosis of FD/MAS-associated FGF23-mediated hypophosphatemic osteomalacia.

Burosumab was initiated subcutaneously at 40 mg, with the subsequent dosing interval adjusted according to serum phosphate. This dose was selected because the patient's body weight was 40 kg at treatment initiation, corresponding to 1.0 mg/kg. Burosumab was started before oral nutritional vitamin D supplementation. Serum phosphate normalized with burosumab alone, without concomitant oral phosphate or active vitamin D analogue therapy. Serial serum phosphate values used for dose and interval adjustment are summarized in Table 1. After the first 40-mg injection, serum phosphate increased from 2.4 mg/dL on the day of injection to 3.8 mg/dL at week 1, but decreased to 2.5 mg/dL by week 5. During subsequent 40-mg injections, 4-week values remained within the reference range, whereas a 5-week value decreased to 2.8 mg/dL. Therefore, the maintenance regimen was set at 40 mg (approximately 1.0 mg/kg based on baseline body weight) at approximately 4-week intervals, with the exact interval individually adjusted between 4 and 5 weeks according to trough serum phosphate.

**Table 1:** Serial serum phosphate levels during burosumab dose adjustment.

	Dosing phase	Body weight	Burosumab regimen	Timing of serum phosphate measurement after the most recent injection	Serum phosphate, mg/dL
Case 1	First injection	40 kg	40 mg (1.0 mg/kg)	Day 0, week 1, week 5	2.4, 3.8, 2.5
	Second injection		40 mg at 5-week interval	Day 0, week 4	2.5, 3.2
	Third injection		40 mg at 4-week interval	Day 0, week 5	3.2, 2.8
	Fourth injection		40 mg at 5-week interval	Day 0, week 4	2.8, 3.4
	Fifth injection		40 mg at 4-week interval	Day 0, week 4	3.4, 3.3
Case 2	First injection	36 kg	30 mg (0.83 mg/kg)	Day 0, week 2, week 3, week 4, week 6	3.1, 6.2, 5.8, 5.4, 3.9
	Second injection		20 mg at 6-week interval	Day 0, week 4, week 6	3.9, 4.6, 3.8
	Third injection		20 mg at 6-week interval	Day 0, week 6	3.8, 3.6
	Fourth injection		20 mg at 6-week interval	Day 0, week 6	3.6, 3.6

Serum phosphate reference intervals were 2.7–4.6 mg/dL for Case 1 (adult male laboratory interval) and 3.7–5.9 mg/dL for Case 2 during the treatment period (2.5th–97.5th percentiles; Pott et al., 2024). Burosumab doses in mg/kg were calculated using body weight at treatment initiation. In Case 2, the initial dose was calculated as 36 kg × 0.8 mg/kg = 28.8 mg and rounded to 30 mg. The dose was reduced to 20 mg and the interval was extended because serum phosphate exceeded the age-appropriate upper reference limit after the initial injection.

Oral nutritional vitamin D supplementation was added thereafter to correct 25-hydroxyvitamin D deficiency. At 11 months after burosumab initiation, serum phosphate was 3.1 mg/dL (2.7–4.6 mg/dL), alkaline phosphatase had decreased to 835 U/L (38–113 U/L), and bone-specific alkaline phosphatase had decreased to 244 μg/L (3.7–20.9 μg/L). No symptomatic treatment-related adverse events were observed during follow-up. Bone pain improved, activities of daily living expanded, and the patient progressed from wheelchair dependence to intensive standing training by 1 year after treatment initiation. No additional fractures occurred during 1 year of follow-up (Table 2).

**Table 2:** Clinical features, diagnostic clues, and short-term outcomes of two cases.

	Case 1: 22-year-old man with FD/MAS	Case 2: 12-year-old boy with FD/MAS
Basis of FD/MAS diagnosis	Polyostotic FD, café-au-lait macules, gonadotropin-independent precocious puberty, and positive *GNAS* testing	Polyostotic FD and café-au-lait macules; *GNAS* testing not performed; no identified MAS-associated endocrinopathy
Initial orthopaedic clue	Recurrent pathological fractures, progressive deformity, loss of ambulation, severe bone pain	Recurrent femoral pathological fractures and impaired growth
Earlier radiographic clue	At age 8, bilateral distal femoral metaphyseal widening, flaring, and fraying with physeal irregularity before first major fracture	At age 5, bilateral distal femoral metaphyseal widening, flaring, and fraying with physeal irregularity before first major fracture
Key diagnostic laboratory data before burosumab therapy	BAP, μg/L: 472 (3.7–20.9) PTH, pg/mL: 85 (10–65) ALP, U/L: 1533 (38–113) P, mg/dL: 2.4 (2.7–4.6) Ca, mg/dL: 9.0 (8.8–10.1) FGF23, pg/mL: 144 (19.9–52.9) 25(OH)D, ng/mL: 5.5 (<20: deficient)	BAP, μg/L: 193 (53.3–156.8) PTH, pg/mL: 80 (10–65) ALP, U/L: 682 (158–525) P, mg/dL: 3.1 (3.7–5.9) Ca, mg/dL: 8.8 (8.8–10.1) FGF23, pg/mL: 82 (19.9–52.9) 25(OH)D, ng/mL: 19.6 (<20: deficient) TmP/GFR, mg/dL: 2.98 (3.9–5.3)
Diagnosis	FD/MAS-associated FGF23-mediated hypophosphatemic osteomalacia	FD/MAS-associated FGF23-mediated hypophosphatemic rickets
Treatment	Burosumab subcutaneous injection, commencing one year prior to the latest follow-up Maintenance: 40 mg every 4–5 weeks	Burosumab subcutaneous injection, commencing seven months prior to the latest follow-up Maintenance: 20 mg every 6 weeks Over-the-counter nutritional vitamin D supplement, 25.0 μg (1000 IU) daily, initiated 2 months after the first burosumab injection
Short-term outcome	Phosphate normalization; pain relief; no subsequent fracture for 1 year; progression to standing training	Improvement in serum phosphate, improved growth velocity, persistently elevated ALP consistent with FD burden, no subsequent fracture for 7 months

*FD,* fibrous dysplasia; *MAS,* McCune-Albright syndrome; *BAP,* bone-specific alkaline phosphatase; *PTH,* parathyroid hormone; *ALP,* alkaline phosphatase; *P,* phosphate; *Ca,* calcium; *FGF23,* fibroblast growth factor 23; 25(OH)D, 25-hydroxyvitamin D; TmP/GFR, tubular maximum reabsorption of phosphate per glomerular filtration rate.

Laboratory results are presented as measured value (reference interval), except for 25(OH)D. Vitamin D deficiency was defined by the Japanese clinical decision limit of 25(OH)D < 20 ng/mL (Okazaki et al., 2017); the 25(OH)D entries therefore show a clinical classification rather than a reference interval. For Case 2, age- and sex-specific published reference intervals were used for phosphate and TmP/GFR (Pott et al., 2024), ALP measured by the IFCC method (Kurihara et al., 2025), and BAP (Wyness et al., 2013). The published BAP interval is assay-specific and is shown as a literature-based comparator. Serum intact FGF23 was measured by CLEIA at SRL, Inc. (Tokyo, Japan); reference interval, 19.9–52.9 pg/mL. For Case 2, serum intact FGF23 (82 pg/mL) was measured at age 12 when serum phosphate was 3.4 mg/dL; the table summarizes key laboratory values obtained during the pretreatment diagnostic evaluation. Burosumab doses in mg/kg were calculated using body weight at treatment initiation.

### 2.2 Case 2: Pediatric FD/MAS with recurrent femoral fractures and rachitic changes

The patient initially presented with right hip pain at age 5, when radiographs obtained at a local clinic revealed a radiolucent lesion in the right femur. He was subsequently referred to a tertiary care hospital, where he was diagnosed with fibrous dysplasia (FD) and managed with observation. At age 7, he presented to our institution for continued follow-up. At that time, a whole-body skeletal survey identified FD lesions involving the skull, right humerus, right femur, right tibia and fibula, and right foot, establishing the diagnosis of polyostotic FD. In conjunction with the presence of café-au-lait macules, these findings supported a clinical diagnosis of FD/MAS; GNAS testing was not performed. Because he remained asymptomatic, annual clinical and radiographic surveillance was performed. At age 9, he sustained a nondisplaced right femoral neck fracture, which was treated conservatively. At age 11, he sustained a pathological fracture of the right femoral shaft and underwent elastic intramedullary nailing. The second fracture prompted further evaluation for MAS-associated endocrinopathies. Endocrine evaluation showed no evidence of MAS-associated endocrinopathy. Specifically, there was no history or clinical evidence of precocious puberty; pubic hair was absent, corresponding to Tanner pubic hair stage 1, and no thyroid dysfunction, growth hormone excess, or gonadal endocrine abnormality was identified.

**Fig. 4:**
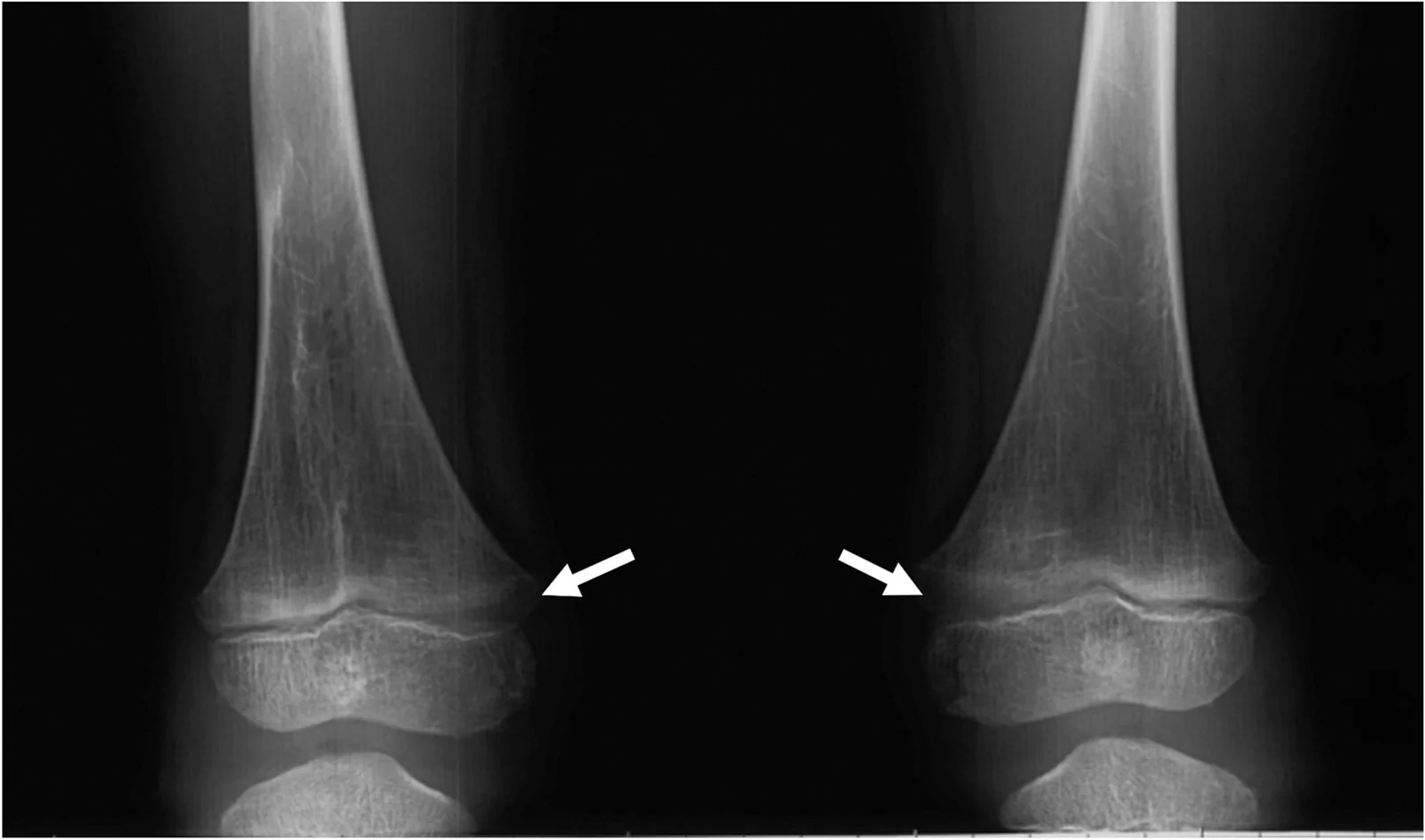
Previously unrecognized rachitic changes in Case 2 at age 5. Anteroposterior radiograph of both knees showing bilateral distal femoral metaphyseal widening, flaring, and fraying, with predominantly medial physeal widening and irregularity. Arrows indicate medial widening and irregularity of the distal femoral physes with adjacent metaphyseal fraying.

**Fig. 5:**
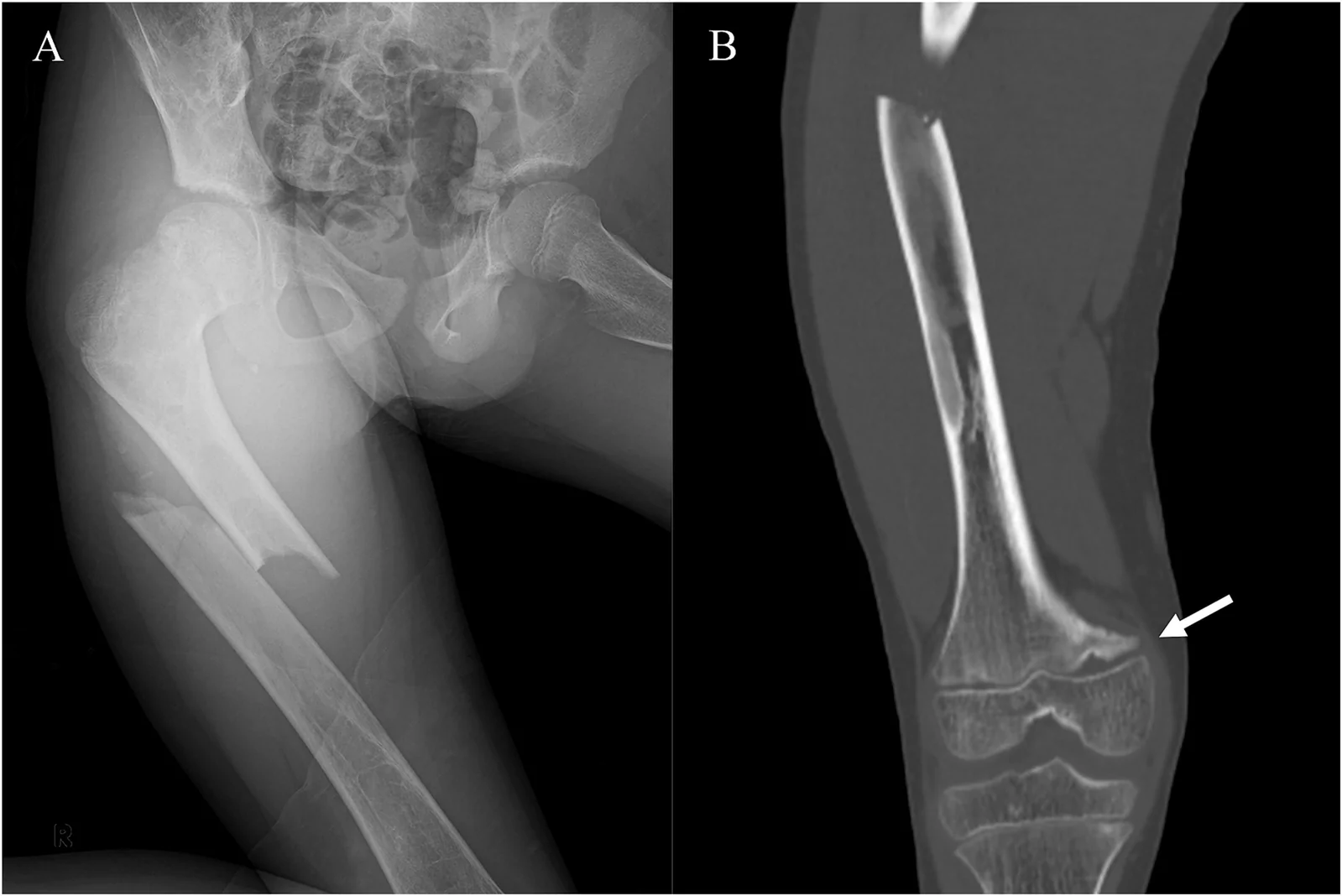
Pathological fracture and persistent rachitic changes in Case 2 at age 11. (A) Anteroposterior radiograph showing a right femoral pathological fracture. (B) Coronal CT image showing distal femoral physeal widening and irregularity with adjacent metaphyseal fraying, compatible with persistent rachitic change. The arrow indicates the distal femoral metaphyseal/physeal abnormality.

During the evaluation at age 11, radiographs obtained at age 5 were retrospectively reviewed and found to show bilateral widening, flaring, and fraying of the distal femoral metaphyses, with predominantly medial physeal widening and irregularity. These findings were consistent with previously unrecognized rachitic changes (Fig. 4**)**. Review of radiographs and CT images obtained at age 11 demonstrated persistent widening and irregularity of the distal femoral physes with adjacent metaphyseal fraying, indicating ongoing rachitic changes (Fig. 5). Given these findings and the recurrent femoral fractures, biochemical evaluation was performed and revealed hypophosphatemia. As part of the diagnostic evaluation for rickets/osteomalacia, serum intact FGF23 was measured at age 12, when serum phosphate was 3.4 mg/dL, and was elevated at 82 pg/mL (laboratory reference interval, 19.9–52.9 pg/mL). At burosumab initiation at age 12.4 years, serum phosphate remained low at 3.1 mg/dL (age- and sex-specific reference interval, 3.7–5.9 mg/dL; Pott et al., 2024). Other pretreatment laboratory findings included alkaline phosphatase 682 U/L (158–525 U/L; IFCC method; Kurihara et al., 2025), bone-specific alkaline phosphatase 193 μg/L (53.3–156.8 μg/L; Wyness et al., 2013), calcium 8.8 mg/dL (laboratory reference interval, 8.8–10.1 mg/dL), intact PTH 80 pg/mL (10–65 pg/mL), and 25-hydroxyvitamin D 19.6 ng/mL (vitamin D deficiency, defined as <20 ng/mL; Okazaki et al., 2017). TmP/GFR was calculated from phosphate and creatinine concentrations measured in simultaneously collected serum and spot urine samples; fasting status and whether the urine represented a second-morning void could not be verified. TmP/GFR was 2.98 mg/dL, below the age- and sex-specific reference interval for 12-year-old boys (3.9–5.3 mg/dL; Pott et al., 2024), supporting inappropriate renal phosphate wasting. The serum phosphate level was also below the Japanese diagnostic threshold for hypophosphatemia even when the more conservative pubertal-to-adult cutoff of ≤3.5 mg/dL was applied. The inappropriately elevated FGF23 level in the setting of hypophosphatemia supported the diagnosis of FD/MAS-associated FGF23-mediated hypophosphatemic rickets/osteomalacia.

**Fig. 6:**
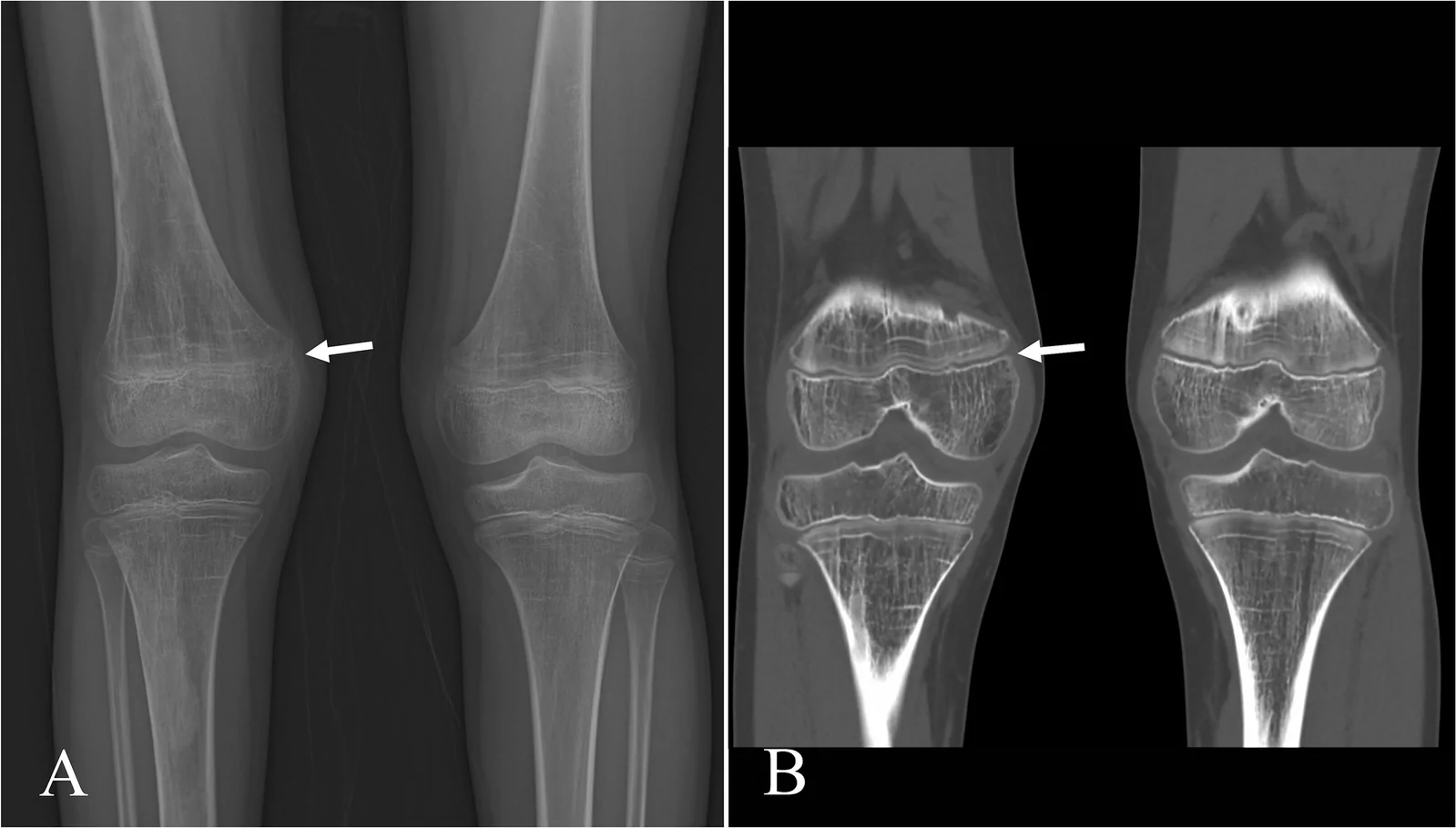
Follow-up imaging of Case 2 six months after burosumab initiation. Anteroposterior radiograph of both knees (A) and coronal CT images (B) show marked normalization of the previously widened and irregular distal femoral physes, with smoother physeal contours and resolution of adjacent metaphyseal fraying. Arrows indicate the medial distal femoral physis.

**Fig. 7:**
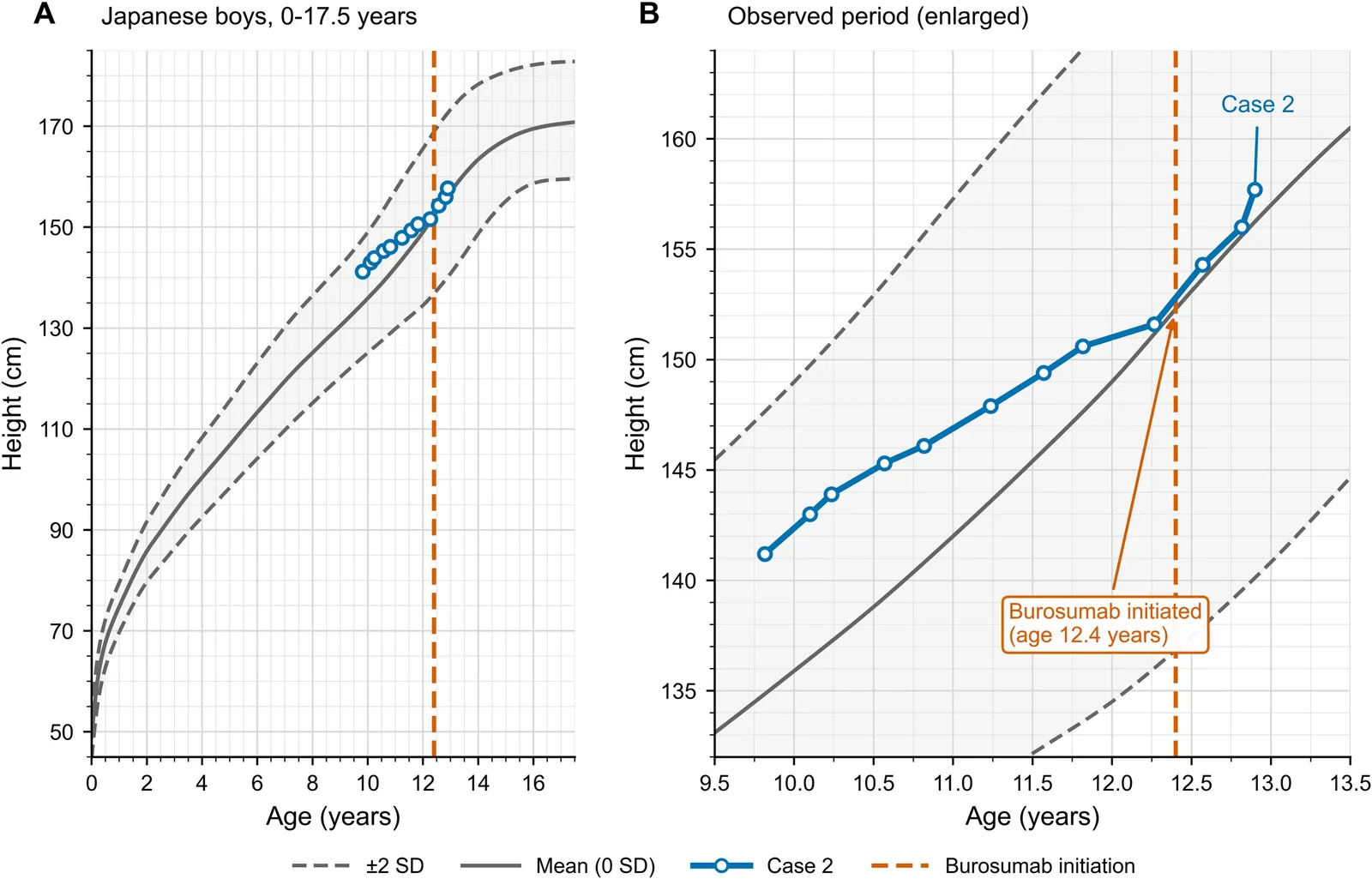
Longitudinal height trajectory of Case 2. Serial height measurements are plotted against the age- and sex-specific mean and ± 2 standard deviation (SD) curves for Japanese boys derived from published LMS parameters (Isojima et al., 2016). (A) Reference chart from birth to 17.5 years. (B) Enlarged view of the observed period. The orange dashed line indicates initiation of burosumab treatment at 12.4 years of age.

Burosumab was initiated at age 12.4 years with a 30-mg subcutaneous dose. This dose was selected by body weight-based calculation: 36 kg × 0.8 mg/kg = 28.8 mg, rounded to 30 mg, corresponding to approximately 0.83 mg/kg. The initially planned dosing interval was every 2 weeks; however, after the first injection, serum phosphate increased from 3.1 mg/dL on the day of injection to 6.2, 5.8, and 5.4 mg/dL during early follow-up. The peak value exceeded the age- and sex-specific upper reference limit of 5.9 mg/dL and indicated transient hyperphosphatemia. The next dose was therefore deferred, the dose was reduced to 20 mg, corresponding to approximately 0.56 mg/kg based on baseline body weight, and the dosing interval was extended to every 6 weeks. Thereafter, serum phosphate values at 6 weeks after subsequent injections were 3.8, 3.6, and 3.6 mg/dL (age- and sex-specific reference interval, 3.7–5.9 mg/dL; Pott et al., 2024), remaining at or slightly below the lower reference limit. The maintenance regimen was set at 20 mg every 6 weeks (Table 1). Burosumab was initiated without concomitant nutritional vitamin D supplementation. After the biochemical response to burosumab alone had been confirmed, the patient began taking an over-the-counter nutritional vitamin D supplement (25.0 μg [1,000 IU] daily) 2 months after the first burosumab injection. Safety monitoring included serial serum phosphate, calcium, and renal function. No symptomatic treatment-related adverse events were observed during follow-up. At 6 months after burosumab initiation, follow-up knee radiographs and coronal CT showed marked normalization of the previously widened and irregular distal femoral physes, with smoother physeal contours and resolution of adjacent metaphyseal fraying (Fig. 6). Growth velocity increased from 3.3 cm/year before treatment to 10.2 cm/year during the follow-up period after treatment initiation; this observation occurred in the absence of clinical precocious puberty or identified MAS-associated endocrinopathy (Table 3; Fig. 7). Alkaline phosphatase remained elevated, consistent with ongoing skeletal FD activity and remodeling. No new femoral fracture occurred during 7 months of follow-up after treatment initiation (Table 2).

**Table 3:** Clinical and biochemical course of Case 2 before and after burosumab initiation.

Date	Treatment phase	Height (cm)	Change from previous measurement	Annualized growth velocity (cm/year)	Serum phosphate (mg/dL)	Serum ALP (U/L)
Jan, X-2		145.3		ND	ND	ND
Apr, X-2		146.1	+0.8	3.2	ND	ND
Sep, X-2		147.9	+1.8	4.3	ND	ND
Jan, X-1		149.4	+1.5	4.5	ND	ND
Apr, X-1		150.6	+1.2	4.8	ND	1039
May, X-1 (Age 11.9)	Surgery for fracture	ND	ND	ND	3.4	909
Sep, X-1		151.6	+1.0	2.4	3.4	916
Nov, X-1 (Age 12.4)	Burosumab initiated	ND	ND	ND	3.1	682
Jan, X		154.3	+2.7	8.1	4.6*	691
May, X		157.7	+3.4	10.2	3.6**	782

*ALP,* alkaline phosphatase, *ND,* not determined.

Age- and sex-specific reference intervals applicable during the period shown were 3.7–5.9 mg/dL for serum phosphate in 11–12-year-old boys (Pott et al., 2024) and 158–525 U/L for ALP in 11–12-year-old boys (IFCC method; Kurihara et al., 2025).

Annualized growth velocity was calculated between consecutive height measurements.

At burosumab initiation, no clinical precocious puberty or MAS-associated endocrinopathy was identified; pubic hair had not developed, consistent with Tanner pubic hair stage 1.

X denotes the year of the latest follow-up; X-1 and X-2 denote one and two years before X, respectively.

*4 weeks after burosumab injection, ** 5 weeks after burosumab injection.

## 3 Discussion

These two cases illustrate an orthopaedic diagnostic pitfall in FD/MAS. Recurrent fractures, deformity, and mobility loss are readily attributed to structurally abnormal dysplastic bone, although FD lesions may also produce excess FGF23, causing renal phosphate wasting and impaired mineralization. In both patients, clinical and imaging clues of defective mineralization led to age-appropriate serum phosphate assessment and then FGF23 testing, following the logic of the 2015 diagnostic flowchart for rickets and osteomalacia (Fukumoto et al., 2015). The key message is not that metaphyseal fraying, physeal irregularity, or low-energy fracture is novel, but that such findings in FD/MAS should prompt biochemical evaluation rather than be ascribed to dysplastic bone alone.

Serial radiographs showed that these clues had preceded metabolic diagnosis by years. Case 1 had bilateral distal femoral metaphyseal changes at age 8, before the first major pathological fracture and subsequent deformity and wheelchair dependence. Case 2 had bilateral distal femoral metaphyseal widening, flaring, and fraying at age 5, and CT at age 11 again showed rachitic change at the distal femoral physis. Because FD itself causes fracture and deformity, mechanical stabilization is naturally prioritized; however, recurrent low-energy fractures, elevated ALP, bone pain, functional decline, growth impairment, or rachitic imaging findings should prompt consideration of FGF23-mediated hypophosphatemic rickets/osteomalacia.

Age-appropriate phosphate interpretation is especially important in children. In Case 2, serum phosphate was low for age at age 12 when FGF23 was measured (3.4 mg/dL) and remained low at burosumab initiation at age 12.4 years (3.1 mg/dL); the applicable age- and sex-specific reference interval was 3.7–5.9 mg/dL at both time points (Pott et al., 2024). Although these values may appear only mildly reduced when adult reference ranges are used, Gun et al. analyzed serum phosphorus with age- and sex-adjusted *Z*-scores and found that both frank hypophosphatemia and low-normophosphatemia were associated with fractures, orthopaedic surgeries, and scoliosis in FD/MAS (Gun et al., 2024). Together with the report by Leet et al. that phosphaturia was associated with earlier and more frequent fractures in polyostotic FD/MAS (Leet et al., 2004), these data support interpreting phosphate in relation to age, skeletal burden, symptoms, and imaging findings rather than as an isolated adult-style laboratory value.

The biochemical findings supported clinically relevant FGF23-mediated disease despite potential confounding by vitamin D/PTH abnormalities. FGF23 excess lowers serum phosphate by reducing renal phosphate reabsorption and impairs vitamin D metabolism by suppressing CYP27B1 and inducing CYP24A1, thereby reducing 1,25-dihydroxyvitamin D activity and promoting vitamin D metabolite catabolism (Riminucci et al., 2003; Jones et al., 2014). In Case 1, severe 25-hydroxyvitamin D deficiency (5.5 ng/mL; deficiency defined as <20 ng/mL) and secondary hyperparathyroidism (PTH 85 pg/mL; laboratory reference interval, 10–65) do not exclude FGF23-mediated hypophosphatemia, because serum phosphate normalized after burosumab initiation and before oral nutritional vitamin D supplementation was started, without concomitant oral phosphate or active vitamin D analogue therapy. This clinical course argues against vitamin D deficiency or PTH-mediated phosphaturia as the sole or dominant cause of hypophosphatemia. In Case 2, 25-hydroxyvitamin D was 19.6 ng/mL, meeting the Japanese criterion for vitamin D deficiency (<20 ng/mL; Okazaki et al., 2017) but only slightly below the decision limit, and PTH was mildly elevated (80 pg/mL; laboratory reference interval, 10–65). The low TmP/GFR in the setting of hypophosphatemia supported inappropriate renal phosphate wasting. In addition, serum intact FGF23 was concurrently elevated when hypophosphatemia was documented at age 12 (82 pg/mL with serum phosphate 3.4 mg/dL). Together, these findings made vitamin D deficiency or PTH-mediated phosphaturia alone insufficient to explain the hypophosphatemia. Coexisting abnormalities in vitamin D/PTH metabolism may nevertheless have contributed to impaired mineralization and should be evaluated and corrected as appropriate.

Burosumab was associated with biochemical improvement in both cases, with case-specific clinical and radiographic improvements. In Case 1, serum phosphate increased into the reference range, ALP and bone-specific ALP decreased, pain improved, activities of daily living expanded, and intensive standing training became possible by 1 year. In Case 2, serum phosphate also improved, follow-up imaging at 6 months showed marked normalization of the previously widened and irregular distal femoral physes, and growth velocity increased in the absence of clinical precocious puberty or identified MAS-associated endocrinopathy. Although this temporal association with growth is clinically relevant, the short follow-up period and the patient's age at treatment initiation preclude attributing growth acceleration solely to burosumab.

Case 2 also illustrates the need for careful phosphate monitoring and individualized dose adjustment. The initial body weight-based dose of 30 mg, approximately 0.83 mg/kg, led to transient hyperphosphatemia, which was controlled by reducing the dose to 20 mg and extending the dosing interval to every 6 weeks. Because no FD/MAS-specific burosumab dosing regimen has been established, treatment should be adjusted according to serial phosphate levels. These biochemical and clinical responses are consistent with recent case reports of burosumab use in FD/MAS-associated FGF23-mediated hypophosphatemia (Sakka et al., 2025; Saito et al., 2026) and with a recent phase 2 open-label trial in patients with FD and FGF23-mediated hypophosphatemia, which reported improvement in phosphate metabolism, reduction in ALP among patients with elevated baseline values, and mobility gains in severely affected children. Importantly, lesion biopsies and ^18^F-NaF PET/CT did not suggest increased FD lesional activity, addressing a key safety concern in treating FD/MAS-associated FGF23 excess (de Jong et al., 2026).

Burosumab should not be interpreted as a treatment for FD lesions themselves. It directly targets FGF23 excess, but the *GNAS*-driven mosaic skeletal disease persists. Dysplastic bone may therefore continue to cause deformity, pain, fracture risk, and elevated ALP even after phosphate metabolism improves. The realistic orthopaedic goal of burosumab in this setting is correction of a superimposed and modifiable mineralization defect that may aggravate skeletal morbidity, rather than reversal of structural FD. The physeal normalization observed in Case 2 should therefore be interpreted as improvement of the superimposed rachitic/mineralization abnormality, not as evidence of reversal of structural FD. Persistent ALP elevation after phosphate correction may therefore reflect residual FD burden and skeletal remodeling, and ongoing orthopaedic surveillance for structural FD remains necessary.

This report has several limitations. It includes only two patients, follow-up is short, and there is no untreated control. Genetic confirmation of FD/MAS was available only in Case 1; Case 2 was diagnosed clinically based on polyostotic fibrous dysplasia and café-au-lait macules, and *GNAS* testing was not performed. Pretreatment TmP/GFR was not available in Case 1. In Case 2, pretreatment TmP/GFR was available, but the diagnosis was primarily supported by hypophosphatemia, inappropriately elevated intact FGF23, and rachitic imaging findings. Post-treatment TmP/GFR values were not used diagnostically because burosumab directly increases renal phosphate reabsorption. Functional and growth-related improvements cannot be attributed solely to burosumab because fracture recovery, rehabilitation, natural clinical change, pubertal timing, and correction of coexisting abnormalities may have contributed. In Case 2, no clinical precocious puberty or MAS-associated endocrinopathy was identified, reducing but not eliminating endocrine or puberty-related confounding.

Despite these limitations, the present cases emphasize that FGF23-mediated hypophosphatemic rickets/osteomalacia is a treatable contributor to skeletal morbidity in FD/MAS that can be overlooked when fractures and deformity are attributed only to dysplastic bone. Because previous case reports have already described burosumab treatment for FD/MAS-associated FGF23-mediated hypophosphatemia, the novelty of the present report is not treatment response alone. Rather, serial imaging demonstrated previously unrecognized rachitic metaphyseal/physeal changes years before biochemical diagnosis, illustrating how FD-related structural abnormalities can obscure a treatable FGF23-mediated mineralization defect. These cases may inform orthopaedic practice by encouraging a combined biomechanical and metabolic approach: in patients with recurrent low-energy fractures, elevated ALP, bone pain, functional decline, growth impairment, or rachitic imaging findings, age-appropriate phosphate assessment and FGF23 testing may allow earlier recognition of a modifiable mineralization defect.

Beyond systemic phosphate wasting, experimental studies suggest that FGF23 may also exert local skeletal effects by suppressing osteoblast differentiation and matrix mineralization, and by promoting pyrophosphate accumulation through auto−/paracrine suppression of tissue-nonspecific alkaline phosphatase (Wang et al., 2008; Murali et al., 2016). Because FD lesions themselves produce FGF23, whether such local FGF23 actions contribute to fragility within *GNAS*-mutated dysplastic bone remains an important but unproven question. Future work should clarify how FGF23 excess, phosphate availability, and *GNAS*-mutated dysplastic bone interact in fracture risk, deformity progression, and healing.

## CRediT authorship contribution statement

**Tomoo Nakagawa:** Writing – original draft, Visualization, Investigation, Data curation, Conceptualization. **Jungo Imanishi:** Writing – review & editing, Writing – original draft, Supervision, Resources, Investigation, Conceptualization. **Ayako Koda:** Writing – review & editing, Investigation, Data curation. **Taisuke Matsuyama:** Writing – review & editing, Investigation. **Kenji Sato:** Writing – review & editing, Investigation. **Asako Yamamoto:** Writing – review & editing, Investigation. **Kentaro Matsui:** Writing – review & editing, Investigation. **Yoshinobu Watanabe:** Writing – review & editing, Supervision. **Hirotaka Kawano:** Writing – review & editing, Supervision.

## Ethics declaration

Written informed consent to publish the article has been obtained from all participants or their legal representatives. The privacy rights of participants have been observed.

This study was performed in compliance with relevant laws, regulatory frameworks and guidelines where the research took place. Ethics committee approval was not required under relevant laws and institutional guidelines. Formal approval from an Institutional Review Board (IRB) or specific regulatory framework is not required for this manuscript. Under the Ethical Guidelines for Medical and Biological Research Involving Human Subjects in Japan and our institutional guidelines, retrospective case reports of standard clinical practice are exempt from formal ethics committee review, provided that patient anonymity is maintained and written informed consent is obtained, both of which have been strictly fulfilled in this report.

This research follows the CARE guidelines and the CARE checklist.

## Ethics statement and consent for publication

Written informed consent for publication of case details and radiographic images was obtained from the adult patient in Case 1 and from the parent/legal guardian of the pediatric patient in Case 2. All clinical information and radiographic images were de-identified, and the signed consent forms are retained by the authors.

## Declaration of Generative AI and AI-assisted technologies in the writing process

During the preparation of this work, the authors used ChatGPT to assist with language editing and manuscript revision. After using this tool, the authors reviewed and edited the content as needed and take full responsibility for the content of the published article.

## Funding

This research did not receive any specific grant from funding agencies in the public, commercial, or not-for-profit sectors.

## Declaration of competing interest

The authors declare the following financial interests/personal relationships which may be considered as potential competing interests: Jungo Imanishi reports a relationship with Kyowa Kirin Co Ltd. that includes: consulting or advisory and speaking and lecture fees. Hirotaka Kawano reports a relationship with Kyowa Kirin Co., Ltd. that includes: consulting or advisory and speaking and lecture fees. Yoshinobu Watanabe reports a relationship with Kyowa Kirin Co Ltd. that includes: speaking and lecture fees. Taisuke Matsuyama reports a relationship with Kyowa Kirin Co Ltd. that includes: speaking and lecture fees. Kenji Sato reports a relationship with Kyowa Kirin Co Ltd. that includes: speaking and lecture fees. If there are other authors, they declare that they have no known competing financial interests or personal relationships that could have appeared to influence the work reported in this paper.

## Data Availability

The data that has been used is confidential.
